# Vascular access-specific health-related quality of life impacts among hemodialysis patients: qualitative development of the hemodialysis access-related quality of life (HARQ) instrument

**DOI:** 10.1186/s12882-020-1683-5

**Published:** 2020-01-14

**Authors:** Robert J. Nordyke, Gina Nicholson, Shawn M. Gage, Ted Lithgow, Jonathan Himmelfarb, Matthew B. Rivara, Ron D. Hays, Karen Woo, John Devin Peipert

**Affiliations:** 1 Beta6 Consulting Group, Los Angeles, CA USA; 2 Humacyte Inc., Durham, NC USA; 3 Division of Nephrology, Department of Medicine, University of Washington, Seattle, WA USA; 4 Department of Medicine, UCLA, Los Angeles, CA USA; 5 Department of Surgery, UCLA, Los Angeles, CA USA; 6 Department of Medical Social Sciences, Feinberg School of Medicine, Northwestern University, Chicago, IL USA; 7 Northwestern University Transplant Outcomes Research Collaborative, Feinberg School of Medicine, Northwestern University, Chicago, IL USA

**Keywords:** Vascular access, Hemodialysis, Quality of life, Qualitative development

## Abstract

**Background:**

End stage kidney disease and hemodialysis dependence are associated with impairments in health-related quality of life (HRQOL), which may be related to vascular access (VA). Few HRQOL measures are VA-specific and none differentiate HRQOL impact by VA type. We developed a VA-targeted HRQOL measure to distinguish the impact of fistulas, grafts and catheters.

**Methods:**

We created an initial item pool based on literature review and then conducted focus groups at 4 US sites with 37 adults and interviews with nine dialysis clinicians about VA’s impact on HRQOL. We then drafted the Hemodialysis Access-Related Quality of Life (HARQ) measure and cognitively tested it with 17 hemodialysis patients. Focus group and cognitive interview participants were diverse in age, gender, years on dialysis, and VA.

**Results:**

We identified six domains for the HARQ: symptoms, physical functioning, emotional impacts, social and role functioning, sleep, and care-related burdens. Cognitive interviews indicated that items were easily understood and supported content validity. Attributing HRQOL impact to VA as opposed to other hemodialysis burden was challenging for some items. Some items were dropped that were considered redundant by patients, limitations while dressing was added, and reference to VA-specific impact was included for each item. The average Flesch-Kincaid reading grade level for the revised 47-item HARQ was 5.3.

**Conclusions:**

The HARQ features VA-specific content not addressed in other HRQOL measures, making it ideal for comparisons of different VA types and new VA technologies. The psychometric properties of the HARQ will be evaluated in future research.

## Background

End Stage Kidney Disease (ESKD) patients on hemodialysis must rely on one of several modes of vascular access (VA), including arteriovenous fistulae (AVF), grafts (AVG), and central venous catheters (CVC). HD patients often refer to their VA as their lifeline, reflecting the centrality of VA to hemodialysis and the impact that VA has on their dialysis experience and health-related quality of life (HRQOL) [1–3]. Patient perspectives on the impact of healthcare delivery on their lives are increasingly seen as key to assessing the value of medical innovations for regulatory and reimbursement decisions [4–6]. In addition to use in clinical trials, HRQOL measures have utility in monitoring patients in dialysis practice, evaluating real-world effectiveness of new treatments, quality measurement programs, and as a factor in choice of VA placement [1, 7, 8]. However, the impact of VA on patients’ health is not fully represented by existing assessment tools [4, 9].

Recent studies have shown that patients rank HRQOL outcomes such as physical functioning, ability to work, and social functioning on par with clinical outcomes such as survival, infections, and hospitalizations [4, 10]. But PROs have been underutilized in dialysis-related research. A systematic review of hemodialysis clinical trials showed that patient-reported outcomes were used very infrequently; only 11% of 168 trials included patient-reported pain measures and only 3% included HRQOL measures [11].

Citing the limited availability of VA-specific HRQOL measures and lack of use in clinical trials, SONG-HD investigators advocated for access function to be a “core measure” in hemodialysis studies due in part to the impact of patient quality of life [9]. To date, the impact of hemodialysis on HRQOL has been based largely on the Kidney Dialysis Quality of Life (KDQOL™) instruments [12], including the KDQOL-36™ [13]. While the impact of VA on HRQOL is included in the KDQOL-36 (1 item) and in the condition-specific VAQ (17 items), the impact of VA on HRQOL in hemodialysis is not well studied [8]. Moreover, HRQOL impacts may differ across different access types and a VA-specific measure could be a valuable tool to assess the patient impact of new VA technologies.

Access-related clinical events, such as infection and thrombosis, are clear drivers of clinical outcomes and costs, and are well differentiated between access types. While the impact on patients of missing a dialysis session in order to have a clot removed and a catheter placed may be obvious, the full patient experience with the VA life-cycle is not well characterized nor included in existing measures. In addition to the time and resource burden of these events, patients also experience burdensome physical symptoms, social impacts, changes in family relationships, and emotional effects all unique to VA that may be distinct from the general process of hemodialysis care [1–3, 13].

The objective of this study was to develop a measure to fully capture and differentiate between patient experiences with AVFs, AVGs, and CVCs as the current standards of care. We aimed to incorporate all relevant HRQOL aspects of the patient experience; namely, patient-reported symptoms, family and social context, and the impact of interactions with the healthcare system. Multiple stakeholders were involved during the development process: clinical practitioners, HRQOL experts, patients and patient advocates.

## Methods

**Fig. 1:**
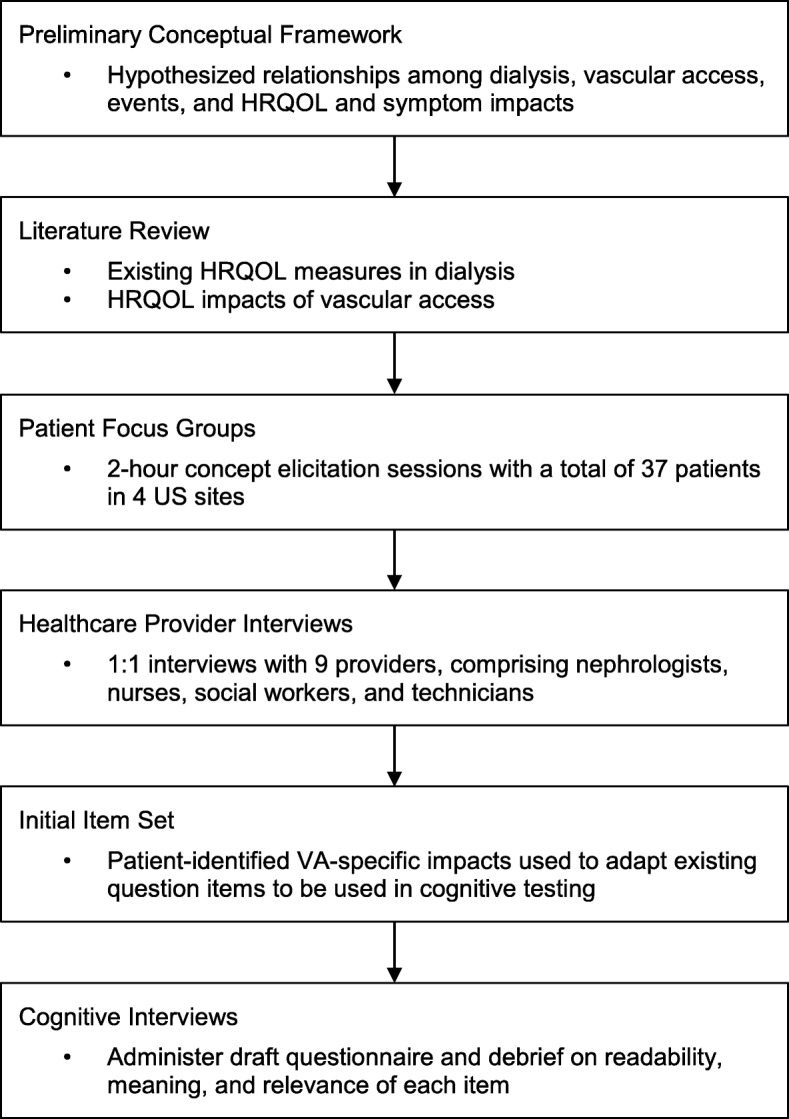
Study Flow

A workgroup of nephrologists, vascular surgeons, instrument development experts, and patient advocates advised during the project (Fig. 1). This team drafted an initial conceptual framework based on experience as patients and healthcare providers, prior published frameworks, and existing instruments addressing ESKD and VA. The draft framework captures the hypothesized relationships among dialysis, VA, events, and HRQOL and symptom impacts and provided overall guidance to subsequent development tasks. The draft framework was revised based on a literature review and, later, results from patient focus groups and healthcare provider interviews. The literature review identified existing studies and instruments related to VA, with the aim of informing the content of the focus groups as well as supplying draft question items for further adaptation post-focus group results. Focus groups were conducted at four sites across the US to elicit impacts of VA as expressed by patients. Provider interviews were then conducted to understand clinicians’ views of the impact of VA on patients. With these inputs, an initial item set was drafted, cognitively tested with patients, and revised. Institutional review board (Ethical & Independent Review Services) approval was granted prior to patient focus groups (#17120–01), HCP interviews (#17177–01) and patient cognitive interviews (#18049-01A). Participants for focus groups, provider interviews and cognitive interviews were identified through the American Association of Kidney Patients (AAKP) and the research arm of DaVita Inc. All focus group (written) and interview participants (oral) provided informed consent.

### Literature review

A literature review was conducted to serve two purposes: 1) identify HRQOL concepts considered in prior research to inform creation of the focus group discussion guide; and, 2) to create a pool of draft items that could be adapted as necessary based on the results of the focus groups. The literature review identified: 1) existing HRQOL measures in dialysis with relevance to VA and 2) studies and review articles focusing on HRQOL concerns related to VA. Multiple MeSH and title/abstract terms were used including: ‘vascular access’; ‘dialysis’; ‘signs’ or ‘symptoms’; and ‘quality of life’ or ‘impact’; and ‘measure’, ‘survey’ or ‘questionnaire’ (search strings included in Additional file 1: Table S1). Articles were included if published in English between 2007 and 2017. Articles were excluded if they reported only on general HRQOL (e.g., SF-36, global health rating) with no VA-relevant HRQOL concepts. In addition, the references of included studies were reviewed to identify additional HRQOL measures that had been published prior to our search timeframe. A single reviewer collected individual question items from included measures and the multidisciplinary workgroup evaluated all items and retained unique items.

### Focus groups

Focus groups were conducted with patients in four sites across the US (Ann Arbor MI, Jacksonville FL, Nashville TN, San Diego CA). Inclusion criteria were: current use of AVF, AVG or CVC, age 18 years or greater, at least 6 months on hemodialysis, conversational English, and ability to participate in 120-min focus groups. Focus group patients were purposively sampled by the American Association of Kidney Patients to be representative of hemodialysis patients in their geographic areas in terms of age, race, and access type. Moderators used a structured interview guide to elicit patient perceptions of the impact of VA on their lives (Additional file 2). Discussions opened with individual reports on history with different types of VA and current VA. This introduction led to a structured discussion of differences in the experiences with AVFs, AVGs, and CVCs, in terms of general perceptions of each type and any unprompted mentions of bother, benefit, or interference. Participants were asked to discuss how their HRQOL is affected by their VA with open-ended questions framed by themes identified in the literature: symptoms, physical functioning, emotional impact, family and social relationships, and ability to work or attend school. Participants were also asked how VA impacts change with the life-cycle of VA, from initial surgical placement, through maturation, successful use for hemodialysis, any infections or thrombotic events, and failure of the access. Focus groups were audiotaped and thematic analysis was conducted on individual responses by 2 independent reviewers and rectified by consensus. New concepts raised in focus groups helped refine the survey item bank.

### Provider interviews

A series of 9 one-on-one web-enabled interviews with clinical professionals (nephrologists, RNs, social workers, dialysis technicians) were conducted to rank symptoms and effects mentioned in the focus group. Providers were also queried on any additional HRQOL impacts that they have encountered during their interactions with patients. Results were coded by 2 reviewers and tabulated.

### Initial item set

The multidisciplinary workgroup reviewed focus group and provider results and identified an initial set of items from published instruments that could be adapted to reflect the impacts expressed by patients in the focus groups as well as the terminology they used. As the focus groups identified impacts that were not represented in existing dialysis measures we also included items from the Patient Reported Outcomes Measurement System (PROMIS®) Physical Function – Short Form 10a and NeuroQOL sets [14]. All items used 5-point polytomous response scales, with the exception of one global pain question using a scale from 1 to 10. Recall periods in the original instruments were used. The initial set of questions comprised 51 items in 6 domains.

### Cognitive interviews

The initial instrument was evaluated with 17 patients, in either web-enabled or in-person interviews. Hemodialysis dependent patients of all educational levels were recruited, with specific focus on patients with high school diploma or less to ensure inclusion of this underrepresented demographic. Respondents were asked to respond to the instrument questions and then queried about readability, meaning and relevance of each question. Participants were geographically diverse and represented all access types. Results were synthesized to refine the instrument.

### Final item set

The workgroup reviewed results from cognitive interviews and edited the instrument for readability and redundancy. We estimated the readability of the final item set using the Flesch-Kincaid scale.

## Results

### Conceptual framework

**Fig. 2:**
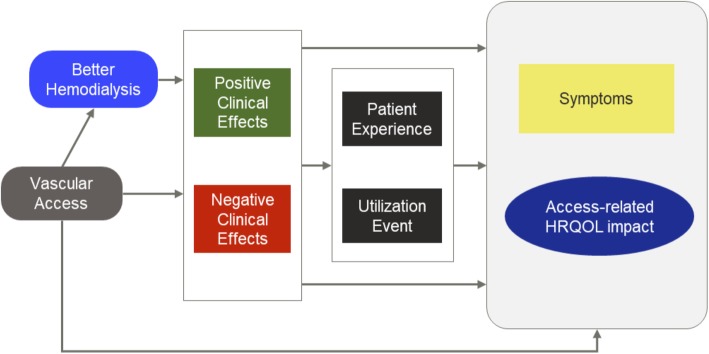
Conceptual framework for symptoms and HRQOL impacts

Multiple pathways by which VA may influence HRQOL were identified, including relationships among disease process, patient factors, signs/symptoms and patient impacts (Fig. 2). Reflecting the complexity of dialysis itself, VA is viewed as affecting patients’ HRQOL and symptoms both directly and indirectly. Key among these are the mediating effects of the patient experience and resource utilization required by adverse events. These relationships were reflected in patient discussions of the challenge in differentiating impacts due to dialysis versus VA specifically as well as the burdens of infections and access-related interventions.

### Literature review

**Fig. 3:**
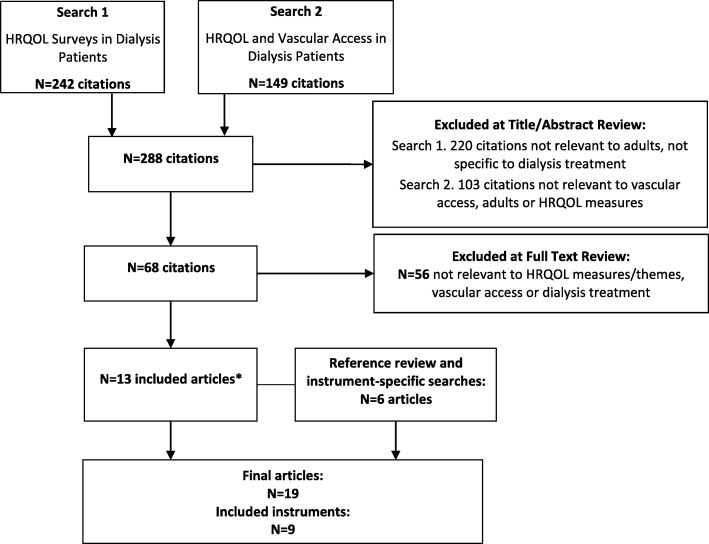
Article Yield from the Literature Review

The review yielded 19 articles of which 9 formal PRO instruments and 2 qualitative studies with patients were identified (Fig. 3 and Additional file 1: Table S2). A set of 300 items representing 15 domains was compiled to form the initial item bank.

### Focus groups

A total of 37 patients participated in the focus groups; key characteristics of these participants are listed in Table 1 and Additional file 1: Table S3. Participants identified worry and fear, physical symptoms, and appearance as the greatest impacts of VA. Example quotes of patient perspectives are listed in Table 2 and Additional file 1: Table S4. Problems with sleep, personal hygiene, and other activities of daily living were also mentioned frequently as burdensome. Needle pain, skin problems, swelling/bruising, and numbness/tingling were expressed as the most burdensome physical symptoms. Personal appearance, particularly clothing choice, and avoidance or awkwardness in social settings were the top social impacts. Several important aspects of VA functioning were commonly cited as affecting HRQOL. Infection risk, cannulation, durability and failure, and interventions required to maintain an access were drivers of emotional impact. Infection risk, maintaining the durability of an access, and needle pain were also mentioned as the reasons for burdensome self-care experiences that patients have while in the dialysis clinic. Concept saturation was reached by the third focus group; no new concepts were voiced by the fourth focus group. Focus group results provided support for the initial conceptual framework. Where focus group results identified effects not fully reflected in the existing item pool, such as mobility and the complexity of social impacts, items were adapted from PROMIS item banks. The full set of domains and impacts as expressed by patients during the focus groups is given in Table 3.

**Table 1:** Table caption

	Focus Groups (*N*=37)		Cognitive Interviews (N=17)	
	*N*	%	*N*	%
Female	21	57%	9	53%
Employed/Student	13	35%	5	29%
Age Group				
25-34	2	5%	2	12%
35-44	2	5%	2	12%
45-54	9	24%	8	47%
55-64	18	49%	3	18%
65-74	5	14%	1	6%
75+	1	3%	1	6%
Race/Ethnicity				
Asian-American Pac Islander	2	5%	0	0%
African American	21	57%	3	18%
Hispanic	2	5%	5	29%
White	12	32%	9	53%
Current Access Type				
AVF	24	65%	10	59%
AVG	10	27%	2	12%
CVC	3	8%	5	29%
	Years		Years	
Years with ESKD				
Mean	8		7.1	
Median	8		4	
Range	27-Jan		0.5 - 21

**Table 2:** Table caption

Selected quotes	Concern / Access type referenced
I'm “terrified because my interventions are so frequent”	Durability / General
It is “always on my mind: will it last, will I run out of places to put an access?”	Durability / General
“It was scary” having that (blood stream) infection	Infections / CVC
You're “waiting for a miracle while it is maturing”	Maturation / General
You “have to make sure that you maintain it and clean it to avoid infection”	Infections / General
I worry about the skills of the techs, they're like an enemy to me	Care at clinic / General
all nurses are different so you need to worry about who to go to and to trust	Care at clinic / AVF
“those needles do not feel good”	Pain / AVG
The “thrill will wake you up”	Sleep / AVF
You can't wear nice clothes and it’s a bummer. No low cut tops	Appearance / General
When I go to parties I don't want to answer the questions	Social interactions / AVF
“A person once told me that I have the 'sign of the beast' on me”	Social interactions / AVF
“I feel weird if I get touched.”	Intimacy / AVF
I “did not like the way it felt when I went running”	Recreation / CVC
“Clots and other interventions just take time, time away from family”	Time burden / AVF
My access “makes it difficult to act as a parent sometimes”	Family interaction / General

**Table 3:** Table caption

Domain	Specific Impact	Number of Mentions	Unique Contribution vs. Existing Dialysis Measures
ADLs / physical function	Housework other ADLs	6	Arm mobility;VA-specific attribution
	Showers / hygiene	9	Mobility related issues; VA-specific attribution
Emotional impact	Fear	16	Access-related infections; VA-specific attribution
	Worry / anxiety	75	Access-related durability; VA-specific attribution
Physical symptoms	Bleeding	5	VA-specific attribution
	Bruising / Swelling	14	VA-specific attribution
	Cramping / Spasms	4	*
	Numbness / tingling	10	*
	Pain at home	7	*
	Pain during dialysis	6	*
	Pain on cannulation	35	VA-specific attribution; patient-centered term “needle-stick”
Sleep	Sleep problems	8	VA-specific attribution
Social / Role Function	Appearance / clothing	33	Inability to wear nice clothes; VA-specific attribution
	Social avoidance/ awkwardness	29	Avoidance and perception of stigma; VA-specific attribution
	Family impacts / play	11	Socializing, family relationships, hobbies; VA-specific attribution
	Work	5	VA-specific attribution
	Physical intimacy	8	VA-specific attribution
	Recreation	10	VA-specific attribution
	Time burden	26	VA-specific attribution
Healthcare interactions	Access-related self-care	20	Burden of self-care
	Vigilance in clinic	29	Burden of self-advocacy

### HCP interviews

A total of nine HCPs (2 technicians, 2 nurses, 3 social workers and 2 nephrologists) participated in the interviews. Results aligned with patient perspectives and supported the conceptual framework; no new impacts were identified by the HCPs. Domains and specific impacts that HCPs consistently rated as “High Impact” to patients included pain on cannulation, time burden and showering / hygiene. Providers also felt that those impacts, in addition to appearance and social impacts, had potential to differentiate between patient experience with different VA types.

### Cognitive interviews

Demographic and clinical characteristics of the patient participants in the cognitive interviews are included in Table 1. Most participants had educational attainment of less than a college degree. The distribution of educational attainment was 29% high school or less; 41% reported some college; and 29% reported a college degree or a higher degree.

Based on review of the cognitive interviews, the workgroup made revisions to the draft questionnaire. Items were dropped due to redundancy if patients reported that questions addressing a common issue were interpreted similarly (e.g., questions about social interactions). Some items were dropped if patients reported difficulty in differentiating the effects of VA specifically from the general impacts of hemodialysis and if there was not a clear clinical rationale linking an impact to VA (e.g., cramping or pain away from dialysis sessions). Questions specific to limitations while dressing were added. The order of questions was changed so that questions with the same recall period (e.g., “…in the last 7 days”) were grouped for clarity. A total of 4 items were eliminated through this process. Reference to VA was added to each question stem to help respondents attribute reported impacts to VA as opposed to hemodialysis. The draft instrument contains 12 question groups (based on recall period) representing a total of 47 items in 6 domains. The Flesch-Kincaid reading grade was 5.3 (range 3.7–6.9).

## Discussion

We used a multi-stage, multiple stakeholder approach to develop a new HRQOL instrument evaluating impacts of VA in hemodialysis. The Hemodialysis Access-Related Quality of life (HARQ) measure was informed by key drivers of impacts reported by a total of 54 patient participants in focus groups and cognitive interviews. The information gleaned from this qualitative work identified the most important impacts reflecting patients’ perspectives. Having elicited this information, we identified the most appropriate items from published HRQOL measures to adapt for inclusion in the HARQ. We followed a patient-centered approach, consistent with FDA guidance for development of PROs [15] which identifies content validity as a critical element of PRO development, established through qualitative research with patients. Content validity is focused on evidence that the items and domains of a PRO instrument are appropriate and are comprehensive relative to its intended measurement concept, population, and use. Moreover, establishing content validity helps determine whether a measure truly captures HRQOL impacts that patients care most about [16]. Our qualitative research demonstrated that the HARQ captures concepts of relevance to dialysis patients and findings were confirmed with practicing nephrologists, dialysis clinicians, and experts in HRQOL research. These included the emotional impacts of VA, impacts on social role function, and physical symptoms. Determining the importance of these issues to patients led to selection of appropriate items for the HARQ.

In addition to concept elicitation, focus group discussions were central to the refinement of the item pool. Items from several previously-published measures were selected and adapted based on patient perspectives on the effects that VA has on their lives. The methodological advantage to sourcing items from prior validated measures (legacy measures), as opposed to writing new items, arises from previously demonstrated understandability and good psychometric properties. Some of these attractive measurement properties can be carried forward even when the items are used in a new subgroup. We tapped the KDQOL-36, VAQ, CHEQ, PROMIS Physical Function item bank, and Neuro-QOL as item sources for the HARQ, all of which are widely used and have strong measurement properties [1, 12–14, 17]. We developed the HARQ using qualitative data from patients to choose the items, and conducted cognitive interviews to determine if any item modifications created barriers to comprehension.

Though items on many important topics were adapted from legacy measures, our qualitative research uncovered important new HRQOL impacts not in these measures. Specifically, issues with mobility and the complexity of the social impacts, including apparent stigma and perceived avoidance of social situations, have not been fully addressed in legacy hemodialysis or VA-specific instruments. A few impacts endorsed strongly by patients have not been fully incorporated into existing instruments but have been highlighted in other qualitative studies on VA in hemodialysis. Based on focus group results and drawing from prior published work, we tested modified items to address the expressed burdens of self-care and patient concerns about the safety of their access during hemodialysis sessions. While it was clear that these issues are perceived as burdens by patients, we anticipate that these items will be captured in a patient experience domain that is separate from the explicitly HRQOL-related issues reflected in other items.

One of our aims was to develop an instrument that differentiates between the access-specific impacts of different VA types. Our results indicate that patients were clearly able to identify impacts that may vary across access types, for example showering problems with a CVC versus AVF or AVG; pain on cannulation with an AVF vs. AVG or CVC. Similarly, we aimed to develop an instrument that can differentiate the impacts of different VA types and to distinguish between impacts from VA and those from dialysis more generally. During the cognitive interviews, hemodialysis patients indicated that they could identify HRQOL impacts that are tied to their VA alone, but that doing so may not be always easy. A deliberate step was taken to indicate this attribution by adding reference to the VA in the items; (e.g., “Worrying about being hospitalized because of problems with your access?”). However, whether the HARQ possesses the ability to detect these differences and which domains may be involved must be evaluated in future analyses.

Psychometric analyses examining this new measures’ reliability, validity, and responsiveness to change in health are needed. These analyses should focus on determining whether the HARQ evidences reliability of at least 0.70 [18] and test-retest reliability of at least 0.40 [19]. These should also test whether there are statistically significant associations at moderate to large magnitudes with similar constructs like overall HRQOL or cumulative symptom burden (convergent validity), lack of associations with constructs that the HARQ should not be related to (discriminant validity), and whether the HARQ indicates worse VA-related complications or other clinical indicators of VA problems are observed (responsiveness). Responsiveness is particularly important for establishing the HARQ’s suitability as an outcome assessment in clinical trials.

Like all research, this study has important limitations to consider. First, while we focused on recruiting a diverse sample of hemodialysis patients from throughout the United States, this sample does not fully represent the national hemodialysis population. Notable omissions from the study sample include Spanish-speaking and pediatric patients. Future work will focus on application and adaptation of the HARQ for these subgroups. Similarly, additional development and validation work would be required for patients in other countries and linguistic groups. Second, as noted above, this paper only covers the development and evidence for content validity of the HARQ, but not does not cover reliability and other types of validity. Though not strictly in the scope of this paper, these measurement properties are important to consider when selecting a PRO instrument. Despite these limitations, the HARQ reflects HRQOL issues that directly relate to the VA that hemodialysis dependent patients have prioritized in importance. The HARQ reflects these issues in a way that is clear and understandable. For this reason, the HARQ may be an appropriate choice of PRO for some applications even before examining other measurement properties in a psychometric validation.

## Conclusions

The HARQ was developed based on extensive qualitative input and feedback from hemodialysis patients and HCPs. Patient participants were representative of an English-speaking US hemodialysis population in terms of hemodialysis vintage, access types and sociodemographic status. The draft HARQ contains 47 items. Future studies are needed to evaluate the psychometric properties of the HARQ. A PRO measure that can differentiate between patient experiences with different VA types will be useful to help identify and to quantify the patient value of novel VA technologies.

## Supplementary information

**Additional file 1 **: **Table S1.** Search Strings: Listing of terms and Boolean logic used for the literature searches**. Table S2.** Existing HRQOL Measures Used as Sources: Existing patient-reported outcome measures and questionnaires included as sources of question item construction for the HARQ. **Table S3.** Prior access among focus group participants: Summary statistics on the number and type of prior access (if any) for hemodialysis patients in the focus groups. **Table S4.** Extended quotes and commentary from focus groups: Additional statements from focus group participants organized by Description of data.

**Additional file 2.** Complete discussion guide for the patient focus groups.

## Data Availability

Data sharing is not applicable to this article as no datasets were generated or analysed during the current study.

## Abbreviations

AAKP: American Association of Kidney Patients
ADL: Activities of Daily Living
AVF: Arteriovenous fistula
AVG: Arteriovenous graft
CVC: Central venous catheter
ESKD: End stage kidney disease
HARQ: Hemodialysis Access-Related Quality of Life
HRQOL: Health-related quality of life
KDQOL™: Kidney Dialysis Quality of Life
PROMIS®: Patient Reported Outcomes Measurement System
SONG-HD: Standardised Outcomes in Nephrology-Hemodialysis
VA: Vascular access
VAQ: Vascular Access Questionnaire
