# Supplementary material for: Vascular access-specific health-related quality of life impacts among hemodialysis patients: qualitative development of the hemodialysis access-related quality of life (HARQ) instrument
Source: BMC Nephrol. 2020 Jan 14;21:16. doi: 10.1186/s12882-020-1683-5 (PMC6958666; doi:10.1186/s12882-020-1683-5)
Supplement: Supplementary file 1 — Additional file 1 : Table S1. Search Strings: Listing of terms and Boolean logic used for the literature searches. Table S2. Existing HRQOL Measures Used as Sources: Existing patient-reported outcome measures and questionnaires included as sources of question item construction for the HARQ. Table S3. Prior access among focus group participants: Summary statistics on the number and type of prior access (if any) for hemodialysis patients in the focus groups. Table S4. Extended quotes and commentary from focus groups: Additional statements from focus group participants organized by Description of data. [file 12882_2020_1683_MOESM1_ESM.docx]

**BNEP-D-19-00781: Supplemental Material**

**Table S1. Search Strings**

|  |  |
| --- | --- |
| *Search 1: Vascular access* | *((((((((((((("vascular grafting"[MeSH Terms] OR "arteriovenous shunt, surgical"[MeSH Terms]) OR "arteriovenous shunt, surgical"[MeSH Terms]) OR "arteriovenous fistula"[MeSH Terms]) OR "arteriovenous fistula"[MeSH Terms]) OR "vascular fistula"[MeSH Terms]) OR "vascular fistula"[MeSH Terms]) OR "catheterization, central venous"[MeSH Terms]) OR "catheters, indwelling"[MeSH Terms]) OR "catheters, indwelling"[MeSH Terms]) OR "central venous catheters"[MeSH Terms]) OR "vascular access devices"[MeSH Terms]) OR "vascular access devices"[MeSH Terms]) OR ("vascular access"[Title/Abstract] OR "AVF"[Title/Abstract] OR "AVG"[Title/Abstract] OR "CVC"[Title/Abstract] OR "fistula"[Title/Abstract] OR "graft"[Title/Abstract] OR "catheter"[Title/Abstract])) AND ("quality of life"[MeSH Terms] AND "renal dialysis"[MeSH Terms]) AND ("humans"[MeSH Terms] AND English[lang]) ((("quality of life"[MeSH Terms] AND "renal dialysis"[MeSH Terms]) AND ((((((((("surveys and questionnaires"[MeSH Terms] OR "surveys and questionnaires"[MeSH Terms]) OR "health surveys"[MeSH Terms]) OR "health surveys"[MeSH Terms]) OR "Surveys and Questionnaires"[MeSH Terms]) OR "surveys and questionnaires"[MeSH Terms]) OR "surveys and questionnaires"[MeSH Terms]) OR "surveys and questionnaires"[MeSH Terms]) OR "surveys and questionnaires"[MeSH Terms]) OR "surveys and questionnaires"[MeSH Terms])) AND ("questionnaire"[Text Word] OR "tool"[Text Word] OR "survey"[Text Word] OR "instrument"[Text Word] OR "domain"[Text Word])) AND ("2007/06/16"[PDat] : "2017/06/14"[PDat] AND English[lang])Search 2: Dialysis-Specific QOL Surveys (Limit 10 years)* |
| *Search 2: Dialysis QOL Tools* | *((("quality of life"[MeSH Terms] AND "renal dialysis"[MeSH Terms]) AND ((((((((("surveys and questionnaires"[MeSH Terms] OR "surveys and questionnaires"[MeSH Terms]) OR "health surveys"[MeSH Terms]) OR "health surveys"[MeSH Terms]) OR "Surveys and Questionnaires"[MeSH Terms]) OR "surveys and questionnaires"[MeSH Terms]) OR "surveys and questionnaires"[MeSH Terms]) OR "surveys and questionnaires"[MeSH Terms]) OR "surveys and questionnaires"[MeSH Terms]) OR "surveys and questionnaires"[MeSH Terms])) AND ("questionnaire"[Text Word] OR "tool"[Text Word] OR "survey"[Text Word] OR "instrument"[Text Word] OR "domain"[Text Word])) AND ("2007/06/16"[PDat] : "2017/06/14"[PDat] AND English[lang])* |

**Table S2. Existing HRQOL Measures Used as Sources**

| **Measure Name** | **Reference Identified** |
| --- | --- |
| CHEQ - CHOICE Health Experience Questionnaire | Wu AW et al. Developing a health-related quality-of-life measure for end-stage renal disease: The CHOICE Health Experience Questionnaire. Am J Kidney Dis. 2001; 37(1): 11-21. |
| DSI – Dialysis Symptom Index | Weisbord SD et al. Development of a symptom assessment instrument for chronic hemodialysis patients: The Dialysis Symptom Index. J Pain Symp Mgt. 2005; 27(3): 226-240. |
| HEMO Quality of Life Assessment | Unruh M et al. Effects of hemodialysis dose and membrane flux on health-related quality of life in the HEMO study. Kidney Int. 2004; 66:355-66. |
| KDQ – Kidney Disease Questionnaire | Laupacis A et al. A disease-specific questionnaire for assessing quality of life in patients on hemodialysis. Nephron. 1992;60(3):302-6. |
| KDQOL-36 | Hays RD et al. Development of the kidney disease quality-of-life (KDQOL) instrument. Qual Life Res. 1994;3(5): 329-38. |
| HSS – Hemodialysis Stressor Scale | Yeh S-C J and Chou H-C. “Coping strategies and stressors in patients with hemodialysis” Psychosomatic Med. 2007; 69:182-190. |
| PROMIS - Patient-Reported Outcomes Measurement Information System | Cella D, et al. PROMIS^®^ adult health profiles: Efficient short-form measures of seven health domains. Value Health. 2019; 22(5): 537-544. |
| PPHS – Patient Perceptions of Hemodialysis Scale | Twomey JC et al. Psychometric properties of the Patient's Perception of Life on Hemodialysis scale. J Nurs Measurement. 2015;23(1):72-81. |
| QASICC - Questionnaire  for Acceptance of and Satisfaction with Implanted Central Venous Catheter | Marcy PY, et al. Patient satisfaction with and acceptance of their totally-implanted central venous catheter: construction and first validation of a questionnaire. J Canc Ther. 2013;5:706-16. |
| VAQ – Vascular Access Questionnaire | Quinn RR et al. The Vascular Access Questionnaire: assessing patient-reported views of vascular access. J Vasc Access. 2008; 9: 122-128. |

**Table S3. Prior access among focus group participants**

| **Prior Accesses** | **N** |
| --- | --- |
| 1, same as current | 4 |
| 2, same as current | 1 |
| Multiple types | 12 |
| No prior | 20 |
| ***Total*** | ***37*** |

**Table S4. Extended quotes and commentary from focus groups**

| **Domain** | **Specific Impact** | **Quotes and summary patient commentary** |
| --- | --- | --- |
| **ADLs / physical function** | Housework other ADLs | "was not able to carry bags when shopping" |
|  | Showers / hygiene | "have to be careful not to get it wet… have to take sponge baths" |
| **Emotional impact** | Fear | I'm "terrified because my interventions are so frequent" |
|  | Worry / anxiety | It is "always on my mind: will it last, will I run out of places to put an access?" |
| **Physical symptoms** | Bleeding | "it's difficult to stop the bleeding" |
|  | Bruising / Swelling | Constant bruising |
|  | Cramping / Spasms | more cramping on a fistula |
|  | Numbness / tingling | Numbness during and after HD |
|  | Pain at home | Having “shooting pain at any time” |
|  | Pain during dialysis | Pain while on machine, during dialysis |
|  | Pain on cannulation | "those needles do not feel good" |
| **Sleep** | Sleep problems | The "thrill will wake you up" |
| **Social / Role Function** | Appearance / clothing | “You can't wear nice clothes and it’s a bummer. No low cut tops” |
|  | Social avoidance/ awkwardness | “When I go to parties I don't want to answer the questions” |
|  | Family impacts / play | My access "makes it difficult to act as a parent sometimes" |
|  | Work | “I was considered 'high risk' at work because I couldn't carry more than 5 lbs with the fistula arm” |
|  | Physical intimacy | "I feel weird if I get touched." |
|  | Recreation | I "did not like the way it felt when I went running" |
|  | Time burden | "These interventions are continual” and take time |
| **Healthcare interactions** | Access-related self-care | You "have to make sure that you maintain it and clean it to avoid infection" |
|  | Vigilance in clinic | worry about the skills of the techs, they're like an enemy to me |
