# Supplementary material for: Vascular access-specific health-related quality of life impacts among hemodialysis patients: qualitative development of the hemodialysis access-related quality of life (HARQ) instrument
Source: BMC Nephrol. 2020 Jan 14;21:16. doi: 10.1186/s12882-020-1683-5 (PMC6958666; doi:10.1186/s12882-020-1683-5)
Supplement: Supplementary file 2 — Additional file 2. Complete discussion guide for the patient focus groups. [file 12882_2020_1683_MOESM2_ESM.pdf]

Hemodialysis Access-Related Quality of Life focus group discussion guide

OVERALL QUESTIONS TO ANSWER IN FOCUS GROUP DISCUSSION:

Note to moderator:
The purpose of this focus group is to determine the following:

- Overall, to understand patient insights and perspective in order to develop an access-sensitive Quality of Life survey
- What do people on hemodialysis think about their experiences with the different ways of receiving hemodialysis?
- What languages do people who need hemodialysis use when discussing the effect of Vascular Access on their lives?
- What Vascular Access-related concerns do people on hemodialysis have?
- How do Vascular Access-related adverse events influence their day-to-day life experiences?
- How do Vascular Access-related issues influence people on hemodialysis' social lives/everyday life?
- What are people on hemodialysis' experiences with Vascular Access and what do they think of the Vascular Access "lifecycle"?

Logistics and Guidance to Group/Lead Statement:

Thank you for coming. The purpose of this meeting is to discuss the how your Vascular Access – your catheter, graft, fistula – makes you feel and how it affects your day to day life. I'm (NAME), and my job is to help facilitate the discussion today**.** As a background, **(***GIVE BRIEF BIO-Where you work, where you are from. Any info that makes you more personal to the group/connects you).*

Today's discussion is called a focus group. For those of you who have never been in a focus group before, a focus group is just a bunch of people getting together to discuss how they feel about a specific topic. Researchers use focus groups to learn more about specific groups of people, and their beliefs, opinions, and feelings on particular topics. In this case, you're all hemodialysis patients. Your feedback will give us valuable information on the effect of different Vascular Access types – catheter, graft, fistula – on your daily life. It is important to note that we do not want to focus on your dialysis sessions or your kidney disease, but rather are concentrating on Vascular Access for this discussion.

As a brief background, we are researchers who have been hired by a firm interested in developing products that could benefit people who need hemodialysis. Today, we will be talking about Vascular Access symptoms, common problems with Vascular Access, as well as the Vascular Access "lifecycle," and how these affect your day-to-day experiences and feelings. When answering the questions, please make sure to distinguish what Vascular Access type you are talking about, or if you are attributing your experience to 'Vascular Access in general'.

There are no right or wrong answers or feelings here; this is an opportunity for you to share your Vascular Access experiences. Everybody is different and has different experiences and feelings. Do not hesitate to speak up when you have a point you would like to make. I'd like the discussion to be informal, so there's no need to wait for me to call on you to respond. In fact, please talk about comments other people make, whether you feel the same way or different. However, during the discussion, it is important to speak one at a time so we can hear everyone's input. If you don't understand a question, please let me know. I am here to ask questions, listen, and make sure everyone has a chance to share. The researchers are interested in hearing from each of you, so if we seem to be stuck on a topic, I may interrupt or change the subject. If I do, please don't feel bad about it, it's just my way of making sure we get through all of the questions and everyone has a chance to talk.

Also, just a few housekeeping items:
First, we would like to ask that everyone keep their cell phones tucked away to avoid distractions during the discussion. The bathrooms are located____.
Please feel free to step out as you need to.

Second, there is a name tag in front of you that we would like you to write whatever name you would like to use for this discussion. You are free to use your real name or a fake name. When you speak during the session if you could state your name before talking that would be great.

Third, we will be tape recording the discussion because we don't want to miss any of your comments. No names will be included in any reports, and researchers will make sure that all information discussed today is confidential. We are also requesting that you make sure personal comments don't leave the room. I hope you'll feel free to speak openly and honestly.

This meeting will last approximately one and a half to two hours. We'll take a ten-minute break about halfway through. You can stop participating at any time or even leave. As a small way of saying thank you, we have some food and drink for you, as well as a $50 gift card.

*(STATE NAME OF NOTETAKER***)** is here to help me by taking notes; s/he may get my attention if I do not see you trying to ask or answer a question. S/he will also be asking some questions throughout the discussion. Let me allow (NAME OF NOTETAKER) to introduce herself.

Hi, I am *(NOTETAKER STATE NAME HERE)***.** As a background, (GIVE BRIEF BIO-Where you work, where you are from. Any info that makes you more personal to the group/connects you).

(STATE NAME OF NOTETAKER 2) will also be taking some notes during the discussion. **(***(STATE NAME OF NOTETAKER 2***),** would you mind introducing yourself?

Hi, I am *(NOTETAKER 2 STATE NAME HERE)*. As a background, (GIVE BRIEF BIO-Where you work, where you are from. Any info that makes you more personal to the group/connects you).

May we turn on the recording?

So now that we've introduced ourselves, I wanted to give you a chance to introduce yourselves. If you don't mind, would you state your first name and how far you drove in today?

It's nice to meet all of you and learn a little bit about you. I think we're now ready to get started. I'd like to start with some background. Going around again, can everyone tell us how long you have been on dialysis, your primary Vascular Access method today as well as, if you have changed your Vascular Access method over time, and if so, what methods have you used?

Thank you!

[Approx. 15 min up to this point]

<u>Discussion Questions</u>

1) To begin, I am going to get your thoughts flowing. When I say Vascular Access, AVF, AVG and/or CVC, what is the first thing that comes to your mind?
    a) PROBES:
        - Can you tell me more about your experiences with your Vascular Access?
        - Do you think about it often?
        - How does it bother or help you?
        - How does it interfere with your daily life?
        - How does it make you feel to have one?

[Approx. 5 min]

2) When I say "FISTULA", arteriovenous fistula or AVF, what is the first thing that comes to your mind?
    a) PROBES as needed:
        - What do you know about AVFs?
        - How do you feel about AVFs?

3) When I say "GRAFT" or arteriovenous venous graft or AVG, what is the first thing that comes to your mind?
    a) PROBES as needed:

- What do you know about AVGs?
- How do you feel about AVGs?

4) When I say “CATHETER”, central venous catheter, or CVC, what is the first thing that comes to your mind?
   a) PROBES as needed:
      - What do you know about CVCs?
      - How do you feel about CVCs?

5) What is the difference to you in using a catheter, fistula and/or graft?
   a) PROBES as needed:
      - Does it make a difference to you whether you are on a catheter, graft or fistula?
      - Do you worry about one of them over the other?

[Approx. 15 min—5 minutes per question]

Some of you discussed this a little bit already, but… I would like to discuss the symptoms of your Vascular Access. How does your Vascular Access make you feel physically?

   b) PROBES as needed:
      - What are the top symptoms that you attribute to your Vascular Access?
      - Can you tell us about a time that you were physically sick from your Vascular Access?
      - Does anything unusual, physically, occur during this time?

6) How does your Vascular Access make you feel?
   a) PROBES as needed:
      - What emotions do you have because of your Vascular Access?
      - Why do you think this made you feel (Use emotion already stated: sad, angry, or frustrated, etc.?)

7) How does your Vascular Access affect your family relationships?
   a) PROBES as needed:
      - What does your family think about your Vascular Access?
      - How has it made you and your family members closer or further apart?
      - How do you think your Vascular Access affects your family?
      - Has it affected family functioning in any way? If yes, how?

8) How has your Vascular Access affected your relationships with others and your social activities?

a) PROBES as needed:
  - What do you think your friends think about your Vascular Access? Neighbors? The mailman?
  - Have your friends treated you differently? If yes, how so?
  - Do you go out more or less with your friends now?
  - Does it prohibit you from particular activities? If yes, how so, and which activities?

9) If you are/were working while on hemodialysis, how has your Vascular Access changed your work-life?
   a) PROBES as needed:
      - How do you think your co-workers think about your Vascular Access?
      - How does it influence your work? Commute? Productivity? Communication?
      - Does it keep you from particular activities? If yes, how so (i.e. going to meetings, going out to dinners)?

10) How does your Vascular Access change your daily activities?
   a) PROBES as needed:
      - How has it changed your daily schedule?
      - In what way has it changed your nutrition in what you cook or eat?
      - How has it changed your daily hygienic habits (i.e. brushing teeth, washing clothes, changing, etc.)?
      - How has it influenced your sleeping habits?
      - What ways has it changed the way you run errands?
      - Have you stopped participating in any hobbies because of your Vascular Access? If yes, what and why?
      - What additional assistance do you need in order to do everyday activities because of your Vascular Access?
      - Have you changed the way in which you manage your home, such as cleaning, gardening, etc.? If yes, how so?

11) Anything we're missing regarding what effect that Vascular Access has had on you?
   a) (Summarize the main issues that arose). Would you say that this is a fair summary of the main points of our discussion around symptoms?
   b) Is there anything that we should add or change?

[Approx. 40 min-50min---10 minute break?]

Welcome back. We've already talked about the effects that your current Vascular Access has on different parts of your lives. I'd like to talk about how you manage your Vascular Access and how you think about living with your Vascular Access.

12) To start, could each of you mention the top thing that you think about your current Vascular Access?
    a) PROBES as needed:
        - What are you worried about?
        - Have you had infections that you can remember? If yes, please expand on how you got the infection. Do you believe that any of these were related to your Vascular Access?
        - Thromboses? Revisions?
        - Skin problems?

13) What were your medical expectations with your Vascular Access, and were they met?
    a) PROBES as needed:
        - If they were not met, what were the differences?
        - What are you worried about?

[Approx. 10-15 min]

14) Okay, we are now going to focus on the Vascular Access "lifecycle." By that I mean the entire experience from learning that you needed surgery, to the surgery itself, to waiting for the Vascular Access to be ready to use, all the way to the point when you may need to have a new Vascular Access. So, can you talk a little about your Vascular Access lifecycle experience from the start to where you are today?
    a) PROBES as needed:
        - How did you make the decision to have surgery for your AVF, AVG or CVC?
        - Were you given any option on which Vascular Access to have? If yes, what did the doctors say?
        - What were your hopes going into the surgery?
        - How have the results from your Vascular Access been compared to your perceived results before the surgery?

15) When discussing maturation with Vascular Access, we mean the time it takes for the fistula or graft to become ready for use. For example, not all accesses are "ready to use for dialysis" right away, some take time to heal or "mature". What does maturation mean to you in terms of AVF and/or AVG?
    a) PROBES as needed:

- How did/or would you feel while it is maturing?
- What are your thoughts if you found out that it failed to mature?
- After it is placed, what are your major concerns? Do you worry about it failing after it is placed?
- What happens if you cannot use it; how do you feel; how does it affect your life?

16) Let's turn to how long your Vascular Access is useable for dialysis and how long it keeps working – some doctors may call that "durability". When you think of your current Vascular Access, how does durability play a role?
    a) PROBES as needed:
        - Is durability something you consider when initially having Vascular Access?
        - How would you feel if you were told that it failed?
        - If it were to fail, what would you do?
        - What do you do to make sure that you can stay on this Vascular Access longer?
        - What is your experience like when you go to the doctor to have your Vascular Access site looked at?

17) We've covered this before, but I want to return to the topic of infections again. Does the possibility of infection influence strongly how you feel about your Vascular Access?
    a) PROBES as needed:
        - What effect do infections have on the time available to you; to your family?
        - Are these something that you worry about in particular?

18) Anything we're missing regarding problems with Vascular Access or the lifecycle?
    a) (Summarize the main issues that arose). Would you say that this is a fair characterization of the main points of our discussion around symptoms?
    b) Is there anything that we should add or change for this particular section?

19) Our last question! Any last comments or questions overall?

[Approx. 30 min]

That concludes our focus group. Thank you so much for coming and sharing your thoughts and opinions with us. If you have additional information that you did not get to say in the focus group, please feel free to contact us – we also have some cards on the table if you'd like to share

any additional information or make any comments. As a reminder, please do not let these personal discussions leave the room. We want to keep your comments confidential. Thank you!
